# Imaginary Worlds and Their Borders: An Opinion Article

**DOI:** 10.3389/fpsyg.2021.793764

**Published:** 2021-12-10

**Authors:** Axel Wiepke, Alex Miklashevsky

**Affiliations:** ^1^Complex Multimedia Application Architectures, Institute of Computer Science, University of Potsdam, Potsdam, Germany; ^2^Potsdam Embodied Cognition Group, Cognitive Sciences, University of Potsdam, Potsdam, Germany

**Keywords:** imaginary world, fiction, narrative, embodied cognition, virtual reality, feeling of presence, mental simulation

In their recent paper “Why Imaginary Worlds?: The psychological foundations and cultural evolution of fictions with imaginary worlds.” (2021), Dubourg and Baumard argue that imaginary worlds - such as those described in The Lord of The Rings, Harry Potter, Star Wars, or Avatar - co-opt our preferences for exploration in the same way as unfamiliar environments in real life do.

Dubourg and Baumard (2021) define worlds as imaginary by using two criteria: a fictional environment described in that world (Section 2 of the original paper) and the uselessness of imaginary worlds for “real” life (imaginary worlds are designed for entertainment only, Section 3). This definition is tautological (worlds are imaginary when they describe fictional, i.e., imaginary environment) and overly limited. This attempted definition forces the authors to exclude from consideration religious narratives (Section 2) explicitly. Moreover, the authors implicitly exclude political and philosophical ideologies, fake news, propaganda, and similar phenomena: all of those, while being fictional, may still refer to the real environment and are certainly not designed for entertainment purposes.

We find the authors' definition of an imaginary world (or fiction) artificially narrow and problematic. Instead, we suggest a more general approach to the definition of “fictional,” which relies on the subjective categorization of a given world as “real” or not: if an individual considers some narrative or the imaginary world as real, it becomes real for that individual and starts influencing their interpretation of the reality, their values, decisions, and choices. Consider the example of Jediism, a novel religion stemming from the fiction novels *Star Wars*. While the world of Jedi and mystic Force remains fictional for many, some individuals accepted it as a real one, so it became real for them. They interpret their reality in line with this narrative and find information from this world, while still fictional for others, essential for their real lives. One more example: whereas for some people, “In the beginning, God created the heavens and the earth.” sounds like the beginning of narrative directly related to the world around them, for others, it does not. Note that the pragmatic decision about the status of the imaginary world comes first (“Is it real or not?”), and only then do individuals decide whether the goal in this imaginary world is mere entertainment or learning something relevant for real life. Our approach makes this sequence of mental events clear. It reduces explanation to the smallest necessary number of entities (message and perceiver). At the same time, Dubourg and Baumard's definition requires an objective comparison between the depicted world and the perceiver's world, implying that there is one more absolute (hidden) authority deciding whether the two correspond.

After re-defining fictional as a subjective category, it is essential to analyze the factors which influence such a categorization process. Why do we decide that some narrative describes the real world? A non-exhaustive list would be (1) specific discursive or pragmatic markers (compare “Once upon a time…” vs. “Breaking news! Our reporters from London…”), (2) correspondence to pre-existing knowledge of listeners, (3) subjective reliability of the source of information, (4) novelty of information (cf. the “mere exposure” effect (Zajonc, 1968), or the “illusory truth” effect in fake news research (Hasher et al., 1977): repetitive information seems more pleasant and trustworthy) and (5) the ease of processing effect (cognitive fluency facilitates non-critical processing of information, e.g., see Newman et al., 2014). In this regard, two particularly interesting phenomena are (6) the feeling of presence observed in virtual reality research and (7) deep sensorimotor simulation during the processing of narratives found in research on embodied cognition.

Research on virtual reality unraveled the so-called “feeling of presence” or “the feeling of being there” (Wirth et al., 2007). Even though a computer-generated environment might not look exactly like a physically possible or aesthetically “real” one, people immersed in it can still perceive this environment as real. To reach this feeling, one needs to pay attention to the medium and locate oneself in the virtual environment. Visual representations of controllers, hands, a whole avatar, or other cues support this process. After building this mental model of the virtual environment, one develops expectations about this world and monitors them constantly. If expectations correspond to actual experiences, physical world disruptions are minimal, and the perceiver accepts the virtual environment as the primary egocentric reference frame, then one feels present. Expectations, in turn, are shaped by numerous factors, such as personality traits and individual characteristics (absorption, age, or gender; Sacau et al., 2008) or cultural logic (values and cultural practices; Papapicco et al., 2021). Therefore, some people accept the virtual environment as “real” while others do not. Furthermore, virtual environments are not for entertainment only since they can be used for training while still not behaving like or representing physically existing surroundings (Huang et al., 2021).

Another vivid example comes from research on embodied cognition. Sensorimotor areas of the brain are active in processing longer narratives (for review, see Meteyard et al., 2012; see also Fischer and Zwaan, 2008; Cayol and Nazir, 2020)—the so-called mental simulation. By exposing their readers to a long narrative, describing physical sensations from different modalities (such as vision, touch, smell, taste, or even interoception–see Connell et al., 2018), the author activates embodied experiential traces in readers' brains as if the readers see, hear or act in the described environment. This immersive experience created by linguistic means might hypothetically also lead to higher acceptance of the narrative as a “real” one.

All the above factors contribute to thinning the subjective border between the “real reality” and fictional reality, as covered by our improved definition of “fictional.” Moreover, our definition is free of tautology and broadens the range of phenomena under consideration. Our approach makes the ability of a world to be “real” measurable: for any given world, a representative sample of participants can evaluate whether they perceive that world as a real or imaginary one. This is an essential advantage that would allow testing Dubourg and Baumard's hypotheses about imaginary worlds experimentally. It also accounts for the fact that the same world may be considered imaginary by some social groups or persons, but not by others.

## Author Contributions

All authors listed have made a substantial, direct, and intellectual contribution to the work and approved it for publication.

## Conflict of Interest

The authors declare that the research was conducted in the absence of any commercial or financial relationships that could be construed as a potential conflict of interest.

## Publisher's Note

All claims expressed in this article are solely those of the authors and do not necessarily represent those of their affiliated organizations, or those of the publisher, the editors and the reviewers. Any product that may be evaluated in this article, or claim that may be made by its manufacturer, is not guaranteed or endorsed by the publisher.
